# Construction of a CNN-SK weld penetration recognition model based on the Mel spectrum of a CMT arc sound signal

**DOI:** 10.1371/journal.pone.0311119

**Published:** 2024-11-25

**Authors:** Wenlong Zheng, Kai Yang, Jiadui Chen, Haisong Huang, Jingwei Yang

**Affiliations:** 1 Key Laboratory of Modern Manufacturing Technology, Ministry of Education, Guizhou University, Guiyang, Guizhou, China; 2 School of Mechatronic Engineering and Automation, Foshan University, Foshan, Guangdong, China; Industrial University of Ho Chi Minh City, VIET NAM

## Abstract

Arc sound signals are considered appropriate for detecting penetration states in cold metal transfer (CMT) welding because of their noninvasive nature and immunity to interference from splatter and arc light. Nevertheless, the stability of arc sound signals is suboptimal, the conventional feature extraction methods are inefficient, and the significance of arc sound attributes for determining penetration statuses is often overlooked. In this study, a compact convolutional neural network (CNN) model is proposed for the adaptive extraction of features from arc sound signals. The model uses the Mel spectrum diagram of an arc sound signal obtained through a short-time Fourier transform (STFT) and a Mel filter bank conversion step as its input. To improve the recognition capabilities of the model, a novel CNN-selective kernel (SK) model for weld penetration recognition is introduced, which integrates the dynamic selection kernel network (SKNet) into the CNN architecture. The experimental results indicate that the CNN-SK model outperforms the traditional models, achieving an accuracy of 98.83% on the validation dataset. This model holds promise for assessing weld penetration in CMT welding applications. The project is available at https://github.com/ZWL58/data/tree/master.

## Introduction

Welding plays a crucial role in various industries. The traditional manual welding methods heavily rely on the theoretical knowledge and practical experience of the welder, leading to potential welding quality inconsistencies. As modern manufacturing technology has continued to evolve, welding has become increasingly intertwined with fields such as machinery, materials, control systems, electrical engineering, and computer science. The progression of welding technology is moving toward automation, intelligence, and digitalization, with the integration of robotics and welding technology offering new opportunities for intelligent welding [1]. This technological advancement has been widely adopted across various industries, including the automotive, aerospace, marine, and ocean engineering fields.

QP980 steel is classified as a lightweight, high-strength steel that offers a superior combination of strength and ductility to that of other steel types. This material exhibits favorable welding characteristics and a strong degree of strength‒plasticity balance [2]. Additionally, QP980 steel is resistant to corrosion from seawater, rendering it suitable for use in high-strength and high-toughness engineering applications, such as submarine pressure shells [3]. Nevertheless, high-strength steel welding poses challenges such as insufficient welding currents, excessive welding speeds, and improper groove angles, leading to issues such as a lack of penetration and excessive penetration. The cold metal transfer (CMT) technique, which is a variation of metal inert gas arc /metal active gas arc (MIG/MAG) welding, addresses these challenges by employing distinct mechanical drop-cutting techniques and offering controlled material deposition through the integration of an innovative wire feeding system and high-speed digital control [4]. CMT has attributes such as a reduced heat input, minimal spatter, low nitrogen content, limited deformation, and a manageable welding process. These features play crucial roles in improving the mechanical properties of welded joints in high-strength steel. To ensure the suitability of high-strength CMT-welded steel joints for practical use in sectors such as the automotive and maritime industries, it is imperative that these joints demonstrate consistently reliable mechanical properties. Consequently, the identification and assessment of the quality of these welded joints are of paramount importance [5].

Conventional approaches for evaluating the integrity of welded joints commonly include visual examinations, mechanical property assessments, chemical composition analyses, nondestructive surveillance, and various other methodologies. Nevertheless, these techniques frequently entail subjective interpretations and are limited to offline execution. Certain monitoring procedures may necessitate sample destruction, thereby compromising the structural integrity of the welded joints. Conversely, assessing weld quality in real time through process data presents advantages such as immediacy, traceability, automation, and intelligence. This facilitates nondestructive online monitoring of all welded joints, representing a pivotal avenue for enhancing weld quality evaluations [6].

CMT welding is a complex physical process that is characterized by multiple parameters, strong coupling, nonlinearity, and time variations. It generates various signals, such as optical, sound, electrical, and thermal signals, each conveying valuable information about the quality of the weld. To establish correlation rules between process information and welding quality, it is crucial to employ multisource information sensing and penetration technology during the arc welding process. This technology uses various types of sensors to monitor and detect multiple welding signals. Each sensor in the utilized measurement and control system can independently measure a specific parameter, and through the use of specialized signal processing techniques, all the independent measurements are combined into a comprehensive measurement value [7, 8]. Multiple sensor systems typically consist of complementary metal-oxide semiconductors (CMOSs) [9], charge-coupled devices (CCDs) [10], microphones [11], Hall sensors [12], infrared thermography [13], spectroscopy [14], etc. These sensors capture various aspects of the welding process, including images of the molten pool; the sound of the arc; the current and voltage of the welding process; the temperature field of the welded joints; and light intensity, wavelength, spectral, and temporal signals. Compared with other sensing methods, sound sensing has several advantages. It offers dynamic responses, is noncontact, and has a low cost. It is not affected by splashing or arc light, making it ideal for data acquisition purposes. Additionally, sound sensing provides strong robustness and dynamic responses.

In CMT welding, the instability and frequency modulation of the arc lead to the generation of arc sounds during the welding process. Psychological experiments have shown that experienced welders can roughly determine penetration states through their senses of hearing [15]. Research [16] has shown that arc sounds can effectively demonstrate the physical process of welding and have great potential for identifying the penetration states of welded joints. To enhance the utilization of arc sound signals, both domestic and international scholars have analyzed these signals from three different perspectives: the time domain, frequency domain, and time-frequency domain. Liu et al. [17] accurate identified penetration states by analyzing time-domain characteristic parameters, such as the short-time energy, average amplitude, average over-zero rate, and zero energy of the observed sound signals. Thekkuden et al. [18] analyzed the frequency-domain characteristics of welding sounds and reported that the peak amplitudes of high-quality welds always exist at a frequency of 216 Hz. Kamal et al. [19] reported that the frequency peak of an auxiliary arc sound varies considerably because of pulse shape changes. They also discovered that frequency peak variations can be utilized to detect welding defects. Although preliminary penetration state identification has been achieved via the time- and frequency-domain feature parameters of sound signals, the universality and accuracy of one-dimensional random signal prediction methods are constrained by the strong noise interference inherent in the welding process. Moreover, the above research heavily relied on manual extraction methods, further limiting the applicability of sound signals in welding defect detection tasks.

The time‒frequency-domain analysis approach combines time and frequency information, allowing for a more comprehensive understanding of the time-varying characteristics of signals. This strategy is highly important for the analysis of nonstationary arc sound signals. Zhao et al. [20] and Ren et al. [21] obtained the time‒frequency spectra of arc sounds via the short‒time Fourier transform (STFT). They reported that wavelet features are more suitable than other signal types for processing arc sound signals. Lv et al. [22] extracted 16-dimensional wavelet features of arc sounds and discovered that the frequency band energy, which is based on wavelet packet decomposition (WPD), is the key factor for recognizing different penetration states. In addition, Mel-frequency cepstral coefficients (MFCCs) [23] were used to extract features from arc sounds. With the benefits of normalizing the energy within each frequency band and providing a higher frequency resolution in the low-frequency region, the Mel spectrum can effectively highlight the significant frequency components in audio signals [24]. The primary focus of the aforementioned study was to extract significant data by compressing the features of sound signals. During the compression process, a considerable amount of the feature information possessed by the original signal may be lost. The Mel spectrum offers a direct approach for analyzing the frequency domain of sound signals, providing an enhanced frequency resolution in the lower frequency range. This capability enables the accentuation of the crucial frequency components within a sound signal. The utilization of Mel-frequency spectra for extracting features from arc sounds has not yet been investigated. Therefore, converting arc sound signals into Mel-frequency spectrograms and extracting new feature parameters from them are innovative ideas.

In addition to the achievements of multisource information sensing, the development and utilization of data-driven, lightweight welding quality recognition models represent crucial technological advancements [25]. With the rapid growth of artificial intelligence, deep learning has emerged as a standout approach because of its superior capabilities in automatic learning and feature extraction tasks. It excels at capturing complex, nonlinear relationships within signals and exhibits strong adaptability and generalizability [26]. Within the broad category of deep learning methods, convolutional neural networks (CNNs) have become widely used techniques because of their ability to leverage large datasets and achieve desired outcomes. A CNN can process data in multiple arrays and extract high-level features via its convolutional, pooling, and fully connected layers. This method is well suited for recognizing various types of nonstructured data and has been successful in diverse fields, including target detection [27], natural language processing [28], pattern recognition [29], and process monitoring [30]. At present, the CNNs used for image classification and recognition mainly include AlexNet, the Visual Geometry Group network (VGGNet), the residual network (ResNet), LeNet, etc. Ji et al. [31] applied the SeCNN-LSTM model to obtain the timing features of strong sound signals and identified and classified arc sound signals. Zhang et al. [32] designed a new 11-layer CNN classification model based on weld images to identify weld penetration defects, and the average classification accuracy reached 99.38%. Liu et al. [33]’s CNN model guided by self-supervised learning (SSL) based on expert knowledge achieved higher recognition accuracy and better interpretability than other methods did in welding ray image recognition tasks. Liu et al. [34] proposed a CNN-LSTM algorithm that combines the advantages of a CNN and an LSTM network, and the test results revealed that the defect detection accuracy was 94%. Jiao et al. [35] proposed associating the weld penetration depth with the arc image information of the penetration pool through a CNN, and the prediction accuracy was improved from 92.7% to 94.2% through this method. The aforementioned research highlights the prevalent utilization of CNNs in welding-based monitoring applications. Nevertheless, these investigations have focused primarily on enhancing the recognition capabilities of models, neglecting considerations regarding model sizes and computational requirements. This may lead to the computational resource demands that are too high, and the subsequent real-time model cannot achieve good protection effects. Hence, methods for obtaining lightweight models and strategies for constructing lightweight welding process monitoring models are needed.

Despite the significant feature extraction progress achieved using deep learning methods based on CNNs, a critical challenge remains: how to effectively capture the essential information contained in images. Attention mechanisms have been introduced to address this problem with the advancement of deep neural networks. Such a mechanism enables a model to selectively focus on and process the critical information contained in the input by assigning weights to different locations or time steps, thereby extracting more meaningful features [36]. An attention mechanism enhances the ability of a CNN to comprehend and model input data by providing spatial and temporal focus. In addition, it is user-friendly and flexible, and it can be easily integrated into any convolutional layer of a CNN. In 2019, Li et al. proposed a selective kernel network (SKNet) that outperformed the existing state-of-the-art architectures in terms of model complexity. The neurons in the SKNet can capture target objects at various scales, adaptively adjust the sizes of their receptive fields based on the input, and demonstrate superior performance [37]. By combining the powerful feature extraction capabilities of the CNN and the dynamic selection mechanism of the SKNet, the effective information contained in the given data is dynamically captured, and excellent recognition performance is achieved.

Currently, the widely used CNN is employed for intricate recognition tasks; this network features a deep network structure and necessitates a substantial volume of data to enhance its recognition performance. Nevertheless, in the domain of welding process monitoring, challenges such as limited data availability and the complexity of model construction impede the application of CNNs. While numerous studies have integrated CNNs into welding applications, the focus has primarily concerned enhancing the accuracy of the developed model rather than considering the model size. Consequently, some models have become excessively deep, requiring more sophisticated equipment. This research aims to address this issue by developing a compact CNN-based penetration recognition network with a lightweight model structure that demonstrates effective recognition capabilities even in cases with limited data. To further enhance its recognition performance, a dynamic selection network is incorporated into the lightweight CNN, resulting in the construction of a CNN-SK recognition model. A comparative analysis with other mainstream CNNs reveals that this model has superior characteristics in terms of its size and parameter count. The empirical findings indicate that the recognition performance of this network surpasses that of traditional models.

The subsequent structure of this article are as follows. The experimental setup and weld penetration state section explains the development of the multisensor platform used for collecting sound signals and presents the experimental design. The arc sound signal dataset construction and characterization section establishes a sound signal dataset, examines the distinctions among the three penetration states from the perspectives of both the time and frequency domains, and elucidates the process of creating the Mel spectrum. The customized lightweight CNN-SK weld penetration state recognition model introduces an enhanced lightweight CNN integrated with SKNet, optimizes the hyperparameters of this network, evaluates the performance of the proposed model in welding experiments, and visualizes the recognition process of the model. Finally, the Conclusions provides concluding remarks.

## Experimental setup and weld penetration state

**Fig 1:**
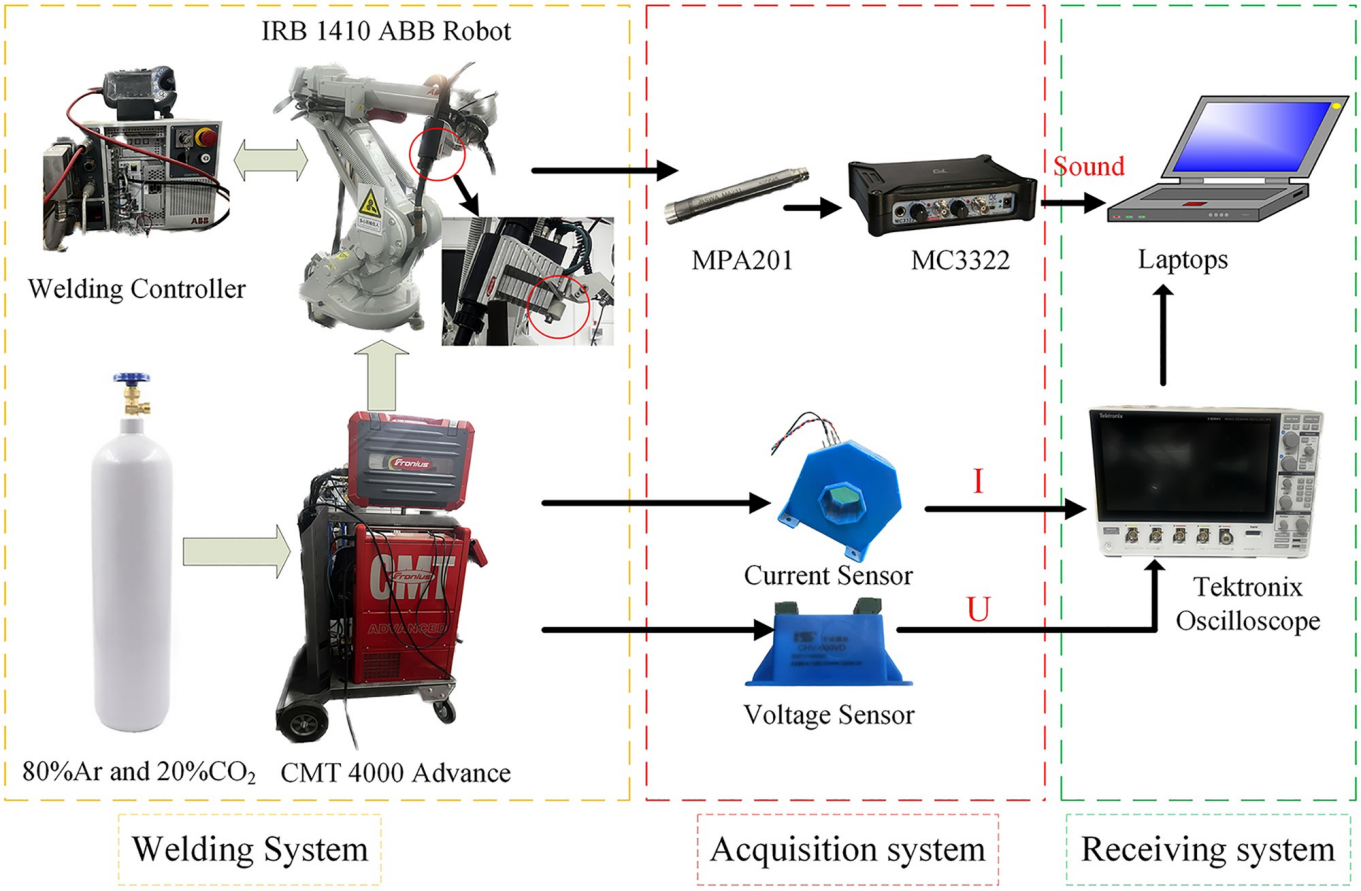
Schematic diagram of the experimental CMT welding platform.

The welding experimental setup utilized in this study is depicted in Fig 1. This setup can be divided into three parts: a welding system, a data acquisition system, and a receiving system. This framework realizes the synchronous acquisition of the welding current, welding voltage, and arc sound during the welding process. The welding system is used to weld high-strength steel, and it is composed of an IRB 1410 ABB robot and a Fronius CMT 4000 Advance additive arc welder. The data acquisition system is mainly composed of information acquisition sensors during the welding process, including an MP201 microphone, a current sensor, and a voltage sensor (particularly an MC3322 signal conditioner for filtering sound signals). The detailed technical parameters of the microphone are displayed in Table 1. During the welding procedure, the torch and microphone remain relatively fixed, with the torch being shifted in a direction perpendicular to the surface of the workpiece while maintaining a consistent distance of 8 mm from the workpiece. Owing to the ignite-exhaust-ignite sequence governing the arc in the CMT welding process, the arc distance undergoes continuous fluctuations.

**Table 1:** The detailed technical parameters of the MP201 microphone.

Type	Frequency response	Dynamic range	Open circuit sensitivity
BSWA MPA201	10 Hz-20 kHz	17–134 dBA	-27.1 dB ± 1 V/Pa(44.2 mV/Pa)

The welding workpiece used in this study is made of a QP980 high-strength steel plate with dimensions of 295 mm × 100 mm × 2 mm. The DC ‘Special 2-Step’ CMT welding process is employed, with a shielding gas mixture consisting of 80% argon and 20% CO2. Throughout the experimental process, the air pressure is maintained at 24 L/min. Given that the welding material is high-strength steel, the same material is selected for the welding wire. Specifically, the ER120S-G high-strength steel wire has a diameter of 1.2 mm. On the basis of insights from the literature [38] and preliminary experimental findings, the process parameters used for the three penetration state experiments are determined as outlined in Table 2, where the current and voltage values of the CMT power supply are bound to the wire feeding speed and cannot be set.

**Table 2:** Welding parameters for the three penetration states.

No.	Robot speed (mm/s)	Wire feeding speed (m/min)	Penetration states
1	4.5	1.8	None penetration
2	4.5	3.2	Full penetration
3	4.5	6.2	Excessive penetration

**Fig 2:**
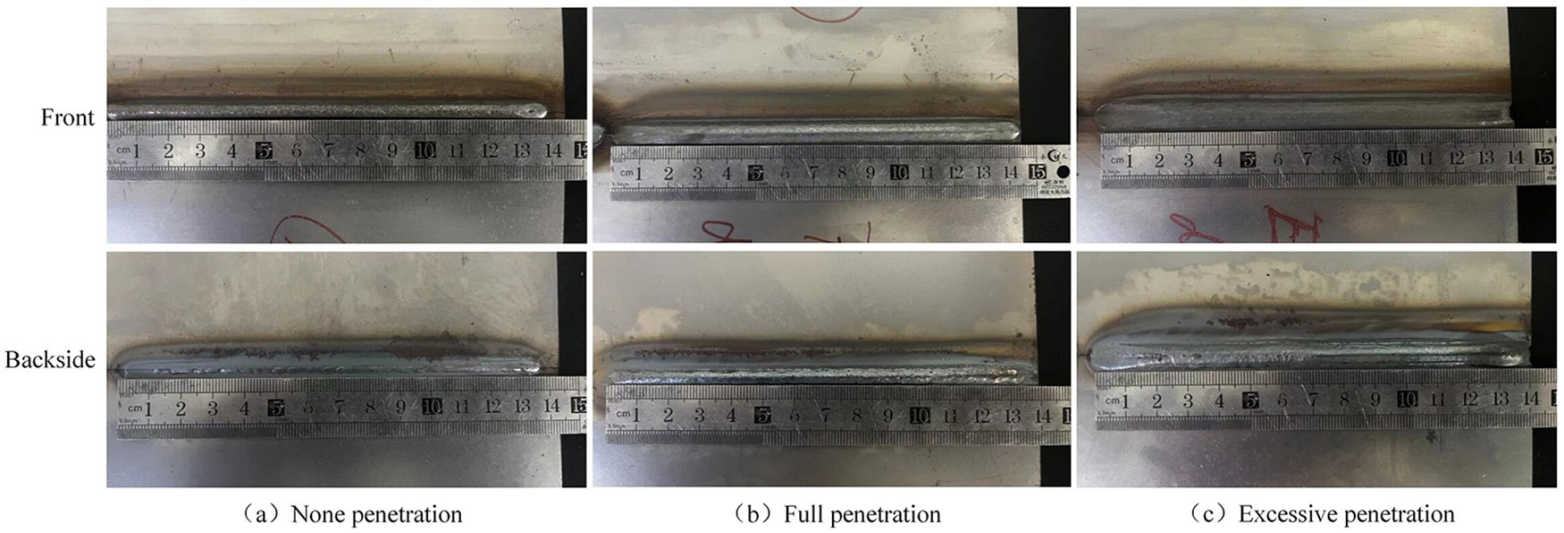
Morphologies of the welded joints in different penetration states.

The surface morphologies of the welded joints in the three penetration-through states described in Table 2 are shown in Fig 2.

## Arc sound signal dataset construction and characterization

### Construction of the sound signal dataset

On the basis of the experimental parameters in Table 2, six experiments are conducted under various experimental parameters to obtain the sound signals of various penetration states. After completing the welding experiments, it is necessary to manually eliminate the unsteady welding and occasional burn-through phenomena that occur in the first 2–3 s at the beginning and end parts of the welding process. This is done to construct a high-quality dataset.

After the improved audio data are acquired, the next step is to convert the one-dimensional time series audio data into two-dimensional image data, which can be used as CNN inputs. First, the arc sound signal needs to be segmented, and the length of each frame is 3000 as an example; refer to reference [39]. Second, the constructed samples are randomly assigned to a training set, a validation set, and a test set at a ratio of 8:1:1, and the distribution of the specific dataset is shown in Table 3.

**Table 3:** Distribution of the dataset.

Penetration states	Train	Validation	Test	Totals
None penetration	800	100	100	1000
Full penetration	800	100	100	1000
Excessive penetration	800	100	100	1000
Totals	2400	300	300	3000

### Time-domain and frequency-domain characteristics of sound signals

**Fig 3:**
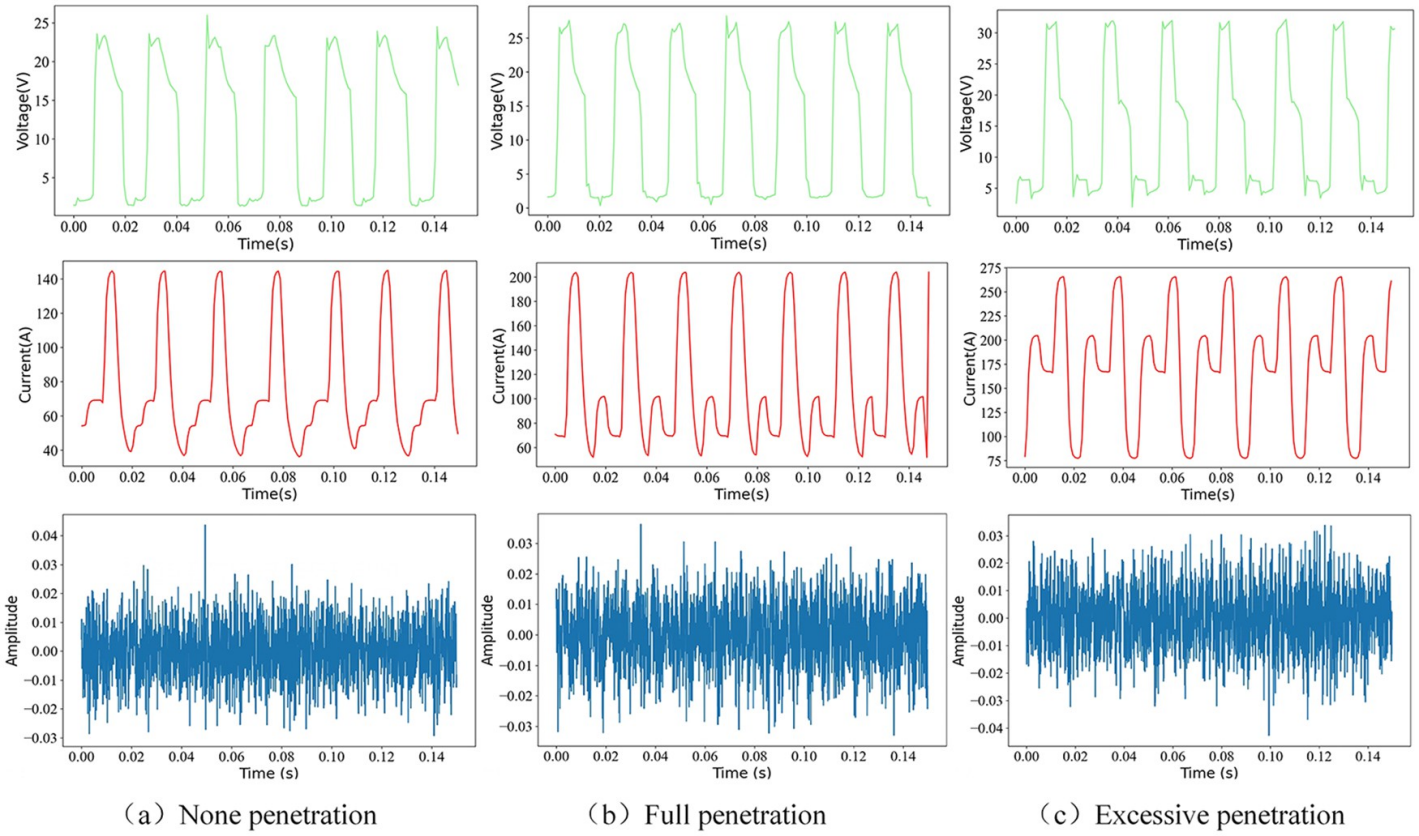
Arc sound signal waveforms and electric signal waveforms of three penetrating states.

Sound signals are unidimensional temporal datasets that depict magnitude variations that occur over time. Fig 3 shows the voltages, currents, and temporal waveforms of arc sound signals across three distinct penetration conditions. During the welding operation, the voltage and current clearly exhibit cyclic fluctuations corresponding to different penetration levels, with notable discrepancies in the waveforms of the voltages and currents among the three penetration states. Conversely, the temporal waveform intensity of an arc sound signal does not display periodic variations, and the temporal waveforms across the three penetration conditions exhibit minimal divergence. This observation suggests that the generation and transmission of arc sounds entail nonlinear propagation, with no discernible disparities among the temporal waveform alterations across the three penetration states.

**Fig 4:**
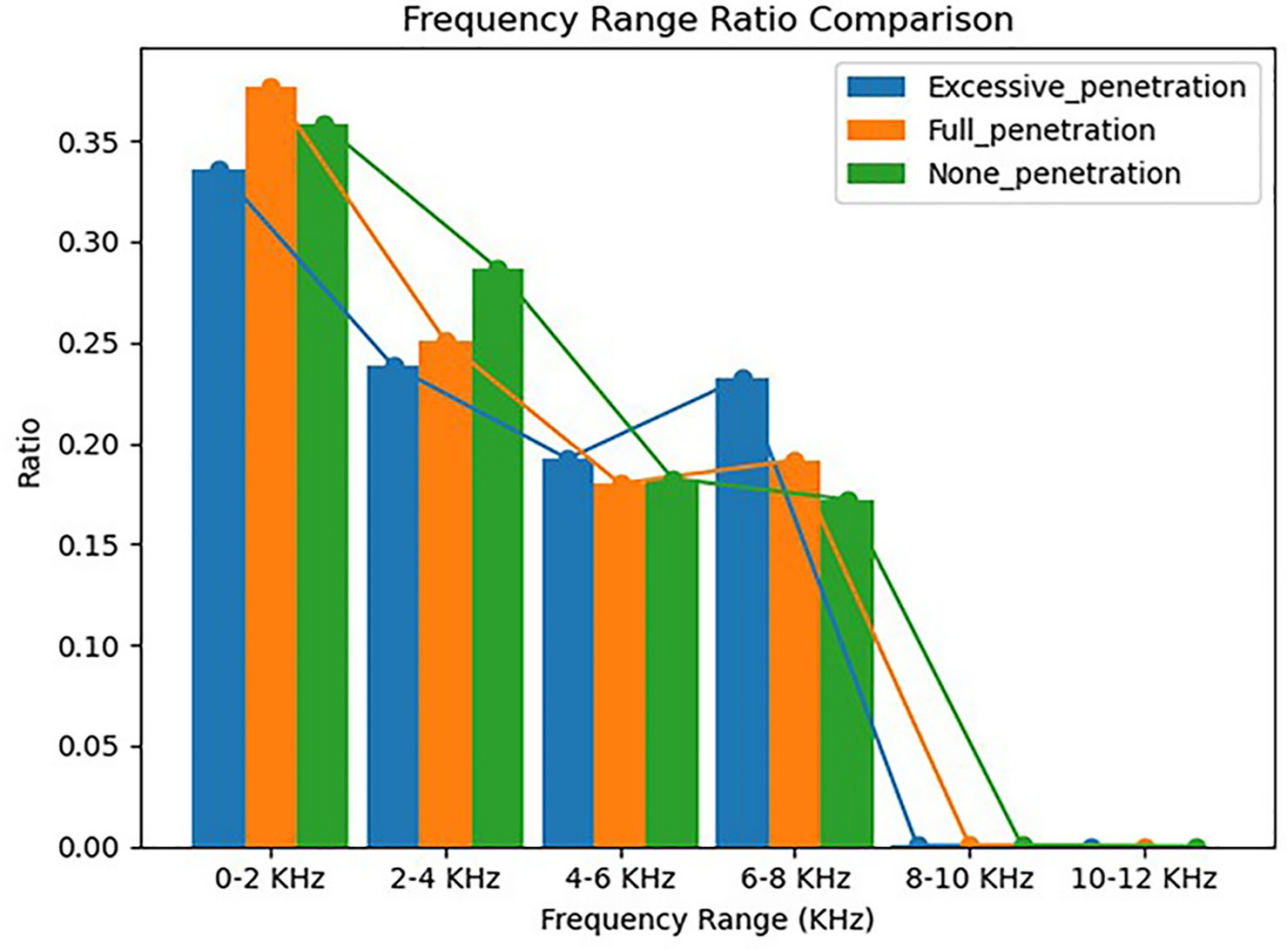
Frequency distributions of the three penetration states.

A random sample is selected from each of the sample datasets representing the three penetration states. The probability distribution of the arc sound frequency is then calculated for each sample within the following intervals: 0–2 kHz, 2–4 kHz, 4–6 kHz, 6–8 kHz, 8–10 kHz, and 10–12 kHz. The frequency distributions of the obtained sound signals are presented in Fig 4. The arc sound frequencies for all three penetration states are primarily concentrated between 0 and 8 kHz, with minimal occurrences beyond 10 kHz. Interestingly, the highest frequency distribution probabilities are consistently observed within the 0–2 kHz interval for all three penetration states. The proportion of the frequency distribution for excessive penetration is slightly greater within the 6–8 kHz interval than within the other two intervals. Compared with the previous distribution, the fully fused state has a slightly higher proportion of its distribution within the 6–8 kHz interval. The unfused state tends toward lower distribution probabilities for higher frequencies. The arc sound signals of the three penetration states demonstrate similar overall frequency components, although local differences that make establishing precise rules for identifying each state challenging.

### Mel spectrum of the sound signals

To compensate for the lack of significant differences among the characteristics of the sound signals in the time and frequency domains, the STFT is used to extend the one-dimensional sound signals to a 2-dimensional time-frequency plane. Arc sound is obtained after the STFT transform, and then, through a Mel filter bank, the sound signals are converted into a Mel-frequency spectrogram. The STFT formula is shown in Eq (1), and the following parameters are used for the STFT: the frame length is set to 1024, the frameshift is set to 768, and the Hanning window is employed.

$$\begin{array}{c} S\left(m,k\right)=\sum_{n=1}^{N-1}x\left(n+mH\right)w\left(n\right)e^{-i2\pi \frac{k}{N}n} \end{array}$$ (1)

where m is the current window, k is the serial number of the current frequency, H is the step size, N is the window size, and w(n) is the window function.

Experienced welders can roughly assess the penetration state of a weld by relying on their senses of hearing. This is attributed to the unique function of the human ear, which allows it to distinguish between different forms of speech even in noisy environments or amidst numerous variations. The cochlea, which is a component of the human ear, essentially operates as a Mel filter bank. Its filtering mechanism operates on a logarithmic frequency scale, with a linear scale below 1000 Hz and a logarithmic scale above 1 kHz. Consequently, the human ear is sensitive to low-frequency signals but less responsive to high-frequency signals. As discussed in Time-domain and frequency-domain characteristics of sound signals, the frequency distributions of the three penetration states predominantly occur within the range of 0–2 kHz. Therefore, the use of a Mel filter bank, which is sensitive to low-frequency signals and relatively insensitive to high-frequency signals, serves as an effective method for extracting the characteristics of sound signals.

The first step toward designing the Mel filter bank is to select the number of filter banks *n*_*mel*_, and *n*_*mels*_ = 64 is selected in this paper. Second, it is necessary to determine the frequency range of the filtering process; usually, the minimum frequency *f*_1_ is 0, and the maximum frequency is Nyquist frequency sr/2. Then, the Hertz (Hz) values are mapped to mel values via the Mel scale in Eq (2), and Mel scales *m*_*l*_ and *m*_*h*_ are obtained for the two frequencies. Afterward, we connect *m*_*l*_ and *m*_*h*_ with a line and then take *n*_*mels*_ points that are equally spaced on this line to obtain a sequence $\left\{m_{1},m_{2},\ldots ,m_{n_{mels}}\right\}$. These points are then converted to Hertz, where the conversion form is shown in Eq (3), yielding the sequence $\left\{f_{1},f_{2},\ldots ,f_{n_{mels}}\right\}$.

$$\begin{array}{c} m=2595\;{\text{log}}_{10}\left(1+\frac{f}{700}\right)=1127\;\text{ln}\left(1+\frac{f}{700}\right) \end{array}$$ (2)

$$\begin{array}{c} \forall f_{i},f_{i}\in \{0,\frac{1}{n_{fft}}S_{r},\frac{2}{n_{fft}}S_{r},\ldots ,\frac{n_{fft}/2}{n_{fft}}S_{r}\},=1,2,\ldots ,n_{mels} \end{array}$$ (3)

**Fig 5:**
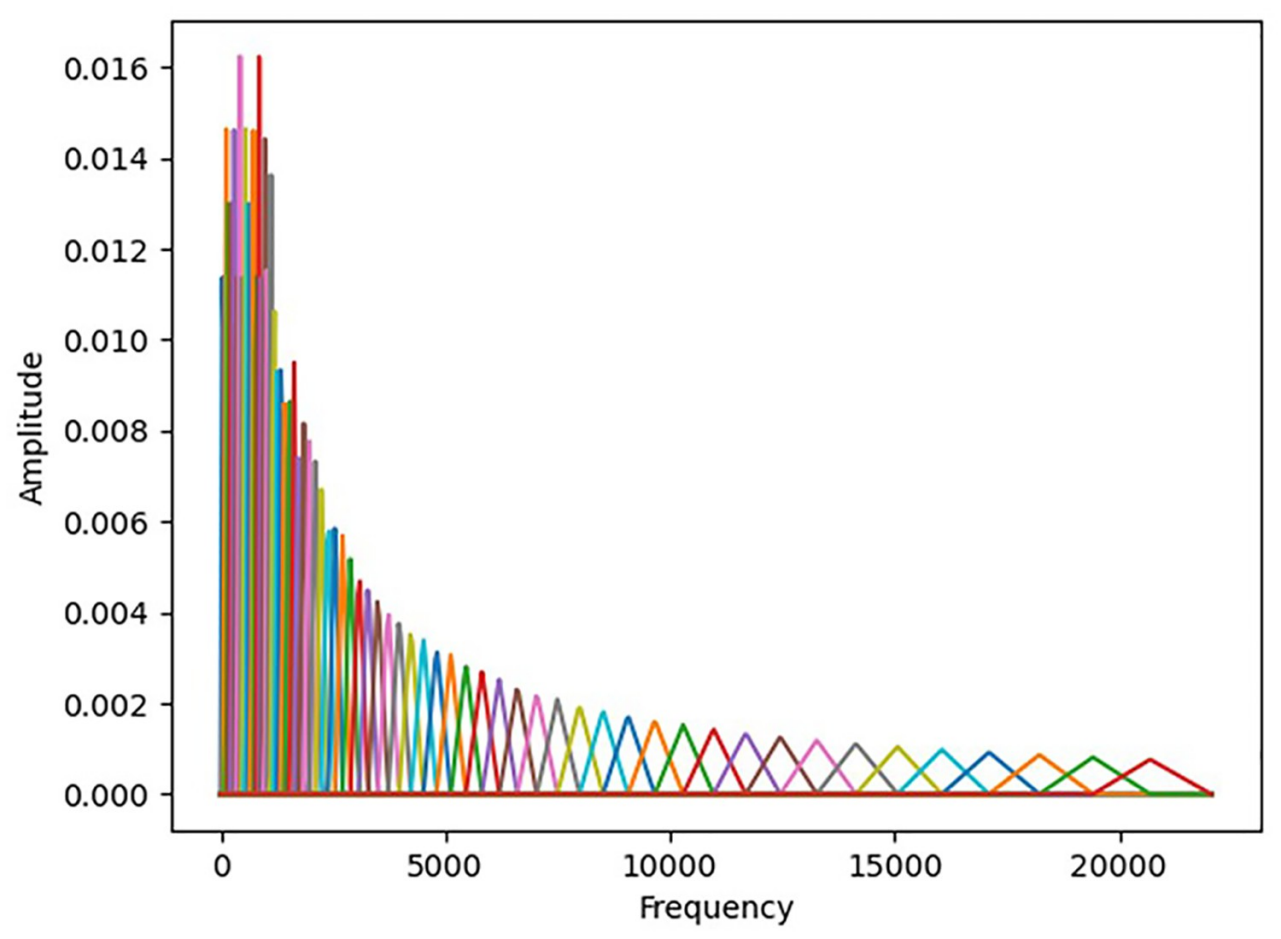
Mel filter bank.

The Mel filter bank drawn according to the sequence $\left\{f_{1},f_{2},\ldots ,f_{n_{mels}}\right\}$ is shown in Fig 5. The graph contains *n*_*mels*_ vertices, and the horizontal coordinate of each vertex follows the sequence $\left\{f_{1},f_{2},\ldots ,f_{n_{mels}}\right\}$. As depicted in Fig 5, a filter bank is created with 64 triangular filters. The filters are densely distributed at low frequencies with a large threshold value, whereas they are sparsely distributed at high frequencies with a low threshold value. This corresponds to the objective law that the higher the frequency is, the later the human ear perceives it. This also reflects the characteristics of the Mel spectrum, which prioritizes low-frequency signals.

**Fig 6:**
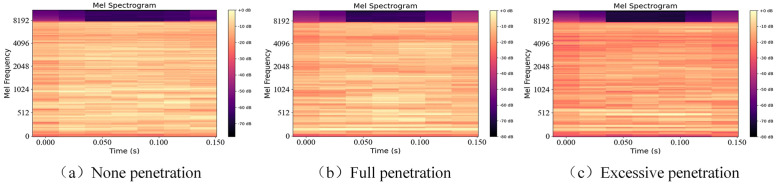
Mel spectra of the sound signals in three penetration states.

The sound signals are filtered via the Mel filter. The amplitude data are subsequently converted into decibels, and the Mel spectrum is generated. Fig 6 displays the Mel spectra of the sound signals corresponding to the three penetration states. The Mel spectra are represented by horizontal stripes, with color shading indicating the intensity levels of the arc sounds at different Mel frequencies. Initially, no significant differences are present among the three penetration states in the high-decibel region. However, during the same period, noticeable distinctions among the color textures of the transverse stripes of the three penetration states can be observed, with the excessive penetration state exhibiting slightly darker colors than those of the other two states. This distinction primarily arises from variations in the frequency distributions of the different penetration states. By further amplifying these subtle differences through filtering via the Mel filter bank, the distinctions between the three penetration states become evident in the form of Mel spectra.

## Customized lightweight CNN-SK weld penetration state recognition model

### Customizing lightweight CNN networks

To enable the three penetration states to be recognized on the basis of the Mel spectra of arc sound signals, a customized CNN model is constructed. The experimental model is implemented via the Python language and PyTorch framework and trained with the following configurations: Windows 10, an Intel i9–12900K CPU, an Nvidia GeForce RTX 3090 GPU, Python 3.8.16, PyTorch 1.12.0, CUDA 11.6.0, and cuDNN 8.5.0. Table 4 summarizes the hyperparameters used for the entire experimental model run. This study uses one-hot encodings to represent the class attributes of images. The research incorporates standard elements found in classification models, such as the cross-entropy loss function, the adaptive moment estimation (Adam) optimizer, and a rule activation function. These three hyperparameters are integrated into the model. The training procedure consists of iterative training for either 50 or 70 epochs, with the initial learning rate and initial batch size established at 0.001 and 32, respectively. Additionally, a learning rate scheduler is implemented to dynamically adjust the learning rate on the basis of a predetermined linear decay strategy.

**Table 4:** Hyperparameter settings.

Parameters	Values
Learning rate	0.001
Optimizer	Adam
Epochs	50, 70
Batchsize	32
Loss function	Cross-entropy loss
Activation function	ReLU

Typically, the current mainstream CNN models, such as VGGNet, ResNet, and AlexNet, are used for more complex tasks and larger-scale datasets. These models consist of multiple convolutional layers and intricate network structures. However, the dataset used in this study is smaller, and the tasks to be processed are simpler. Utilizing a highly complex model may prove counterproductive. Consequently, a lightweight CNN with a simplified network architecture is developed for this study. It has high training efficiency and is better suited for training on small-scale datasets.

**Fig 7:**
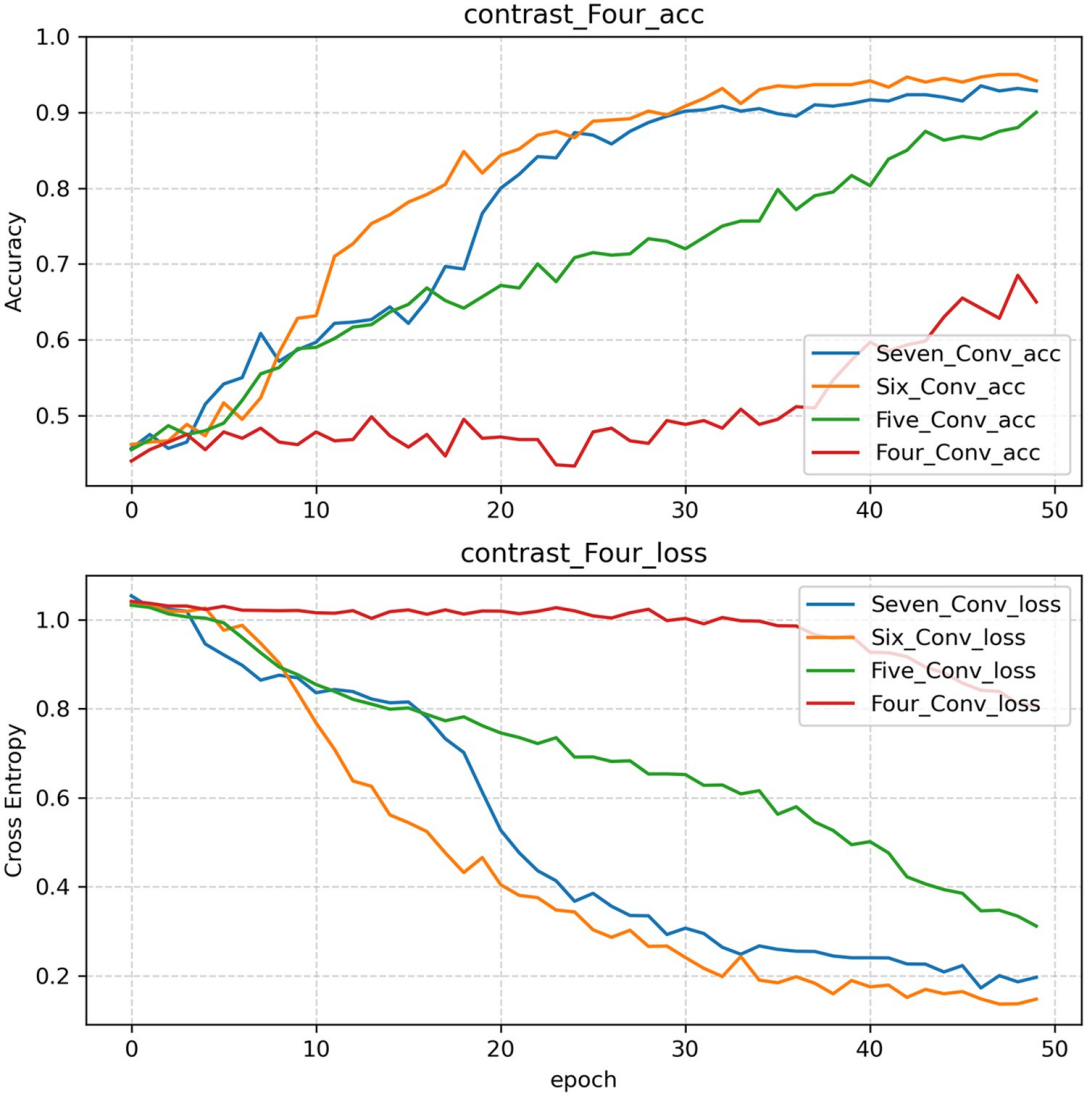
Accuracies and losses produced with different numbers of convolutional layers during the validation process.

The CNN proposed in this paper consists primarily of convolutional and normalization layers. Pre-experiments indicate that selecting only 3 convolutional layers results in low recognition efficiency and accuracy. On the other hand, selecting 8 layers of convolution results in excessively long running times, as the computational workload increases exponentially with additional layers. This contradicts the original intention of designing a lightweight network. To ensure that high accuracy is achieved when recognizing penetration states while maintaining a reasonable training time, the comparison focuses on models with 4, 5, 6, and 7 convolutional layers. The accuracy and loss curves produced by the models during the validation process are shown in Fig 7, and a performance comparison among the models is presented in Table 5. The figure and table clearly show that the training time of model 4 is the longest, whereas the training accuracy of model 1 is relatively low. The training accuracy of model 3 is the highest, and its training time and convergence speed are better than those of the other models. Therefore, model 3 is the most appropriate benchmark model for this paper.

**Table 5:** Performance comparison among models with different numbers of convolutional layers.

Model	Convolutional layers	Accuracy (%)	Training time (epoch = 50)
1	Four layers	65.00	5 min 07 s
2	Five layers	90.00	4 min 34 s
3	Six layers	94.17	4 min 53 s
4	Seven layers	92.83	6 min 23 s

**Fig 8:**
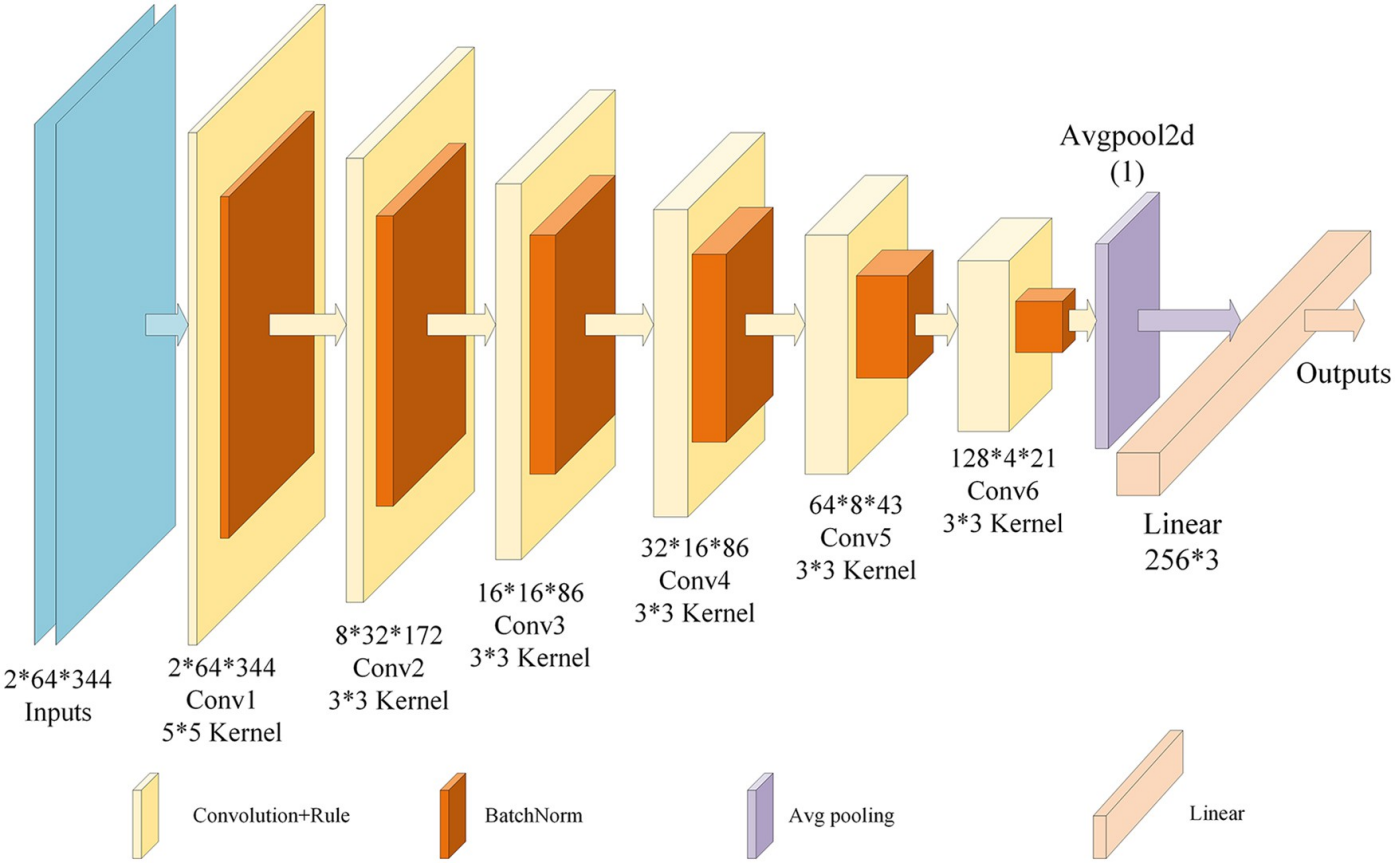
Structure of a custom lightweight CNN.

The overall structure of the model is determined as shown in Fig 8, and the network consists of six convolutional layers (Conv), six normalization layers, an average pooling layer, and a fully connected layer (FC). The convolutional layers are used mainly for feature extraction, the normalization layer is used mainly to combat gradient vanishing, the average pooling layer is used mainly for parameter approximation, and the fully connected layer is used for the final classification step.

The convolutional layer functions similarly to a filter, but the convolution operation overcomes the limitations of traditional filters. It enables local sensing and parameter sharing, facilitating feature extraction on the basis of the chosen objective function. However, the data distribution may change after linear multiplication and nonlinear processing occur in the convolutional layer. As a deep network undergoes multiple layer operations, the data distribution can change even further, which may result in slow learning or gradient vanishing, leading to training stagnation. Normalization serves as an effective solution for protecting against gradient vanishing. By adding normalization operations after each convolutional layer and activation function, the data distribution consistently remains within a range that is sensitive to random changes. This eliminates the need to consider data distribution changes, thus enhancing the effectiveness of training. After multiple convolution and normalization layers, the number of parameters in the data increases significantly. To address this issue, the inclusion of an average pooling layer after the convolutional layers helps reduce the number of network parameters and decreases the spatial size of the data. This not only minimizes the computational resource consumption level of the model but also effectively controls overfitting and improves the generalizability of the model. Additionally, the average pooling layer preserves the scale of the remaining information while expanding the receptive field, which enables more efficient training. Connecting the average pooling layer to the fully connected layer ensures that the scale of the remaining information remains unchanged. Simultaneously, it expands the receptive field, facilitating better image characteristic expressions. The fully connected layer transforms all feature matrices acquired from the average pooling layer into 1-dimensional feature vectors, which are then used for classification in the CNN. Since the convergence speed of the model is average when six convolutional layers are employed, this paper proposes embedding the SKNet based dynamic selection mechanism into the custom CNN to achieve improved recognition penetration.

### Dynamic selection kernel network

When training CNN models, all the data features are treated equally, leading to resource waste and slower model performance. However, if a network can automatically extract information via receptive fields that are suitable for classification purposes, with varying levels of attention paid to receptive fields with different sizes and targets with different scales, the performance of the model can be significantly improved. In deep learning, the ability to focus on the key parts of the input data is known as attention. SKAttention is a new attention mechanism that dynamically selects convolutional kernels for effectively extracting essential information from the input data. This approach is inspired by the fact that humans adjust the sizes of the receptive fields in their visual cortices based on the stimuli encountered when observing objects with different sizes and distances. This mechanism enables each neuron to adaptively adjust the size of its receptive field on the basis of the multiple scales of the input information, so it is referred to as a “selective kernel.” Consequently, it efficiently captures multiscale features in complex image spaces.

**Fig 9:**
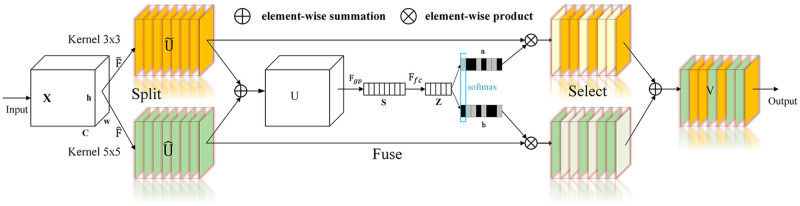
SKNet framework.

Inside SKAttention, the main component is a building block called a selective kernel (SK), which incorporates multiple branches with varying kernel sizes. Guided by the information derived from these branches, information propagation is performed via the SoftMax function. The SKNet consists of multiple SK units, and the SKNet involves three key operations: splitting, fusion, and selection. The structure of the SKNet is illustrated in Fig 9 (only two branches are shown). The splitting operator generates multiple paths with different kernels, whereas the fusion operator combines and aggregates the information derived from these paths to obtain a global and integrated representation that is used to select weights. The selection operator reaggregates the feature maps of kernels possessing different sizes via selection weights.

Theoretically, the SKNet can be incorporated into any two convolutional layers of a CNN. However, the actual improvement provided by the model is subject to debate. Owing to the positioning of the attention mechanism within the backbone, the pretraining weights of the network are not utilized. Therefore, this paper applies the attention mechanism to the feature extraction network to maximize its benefits.

### Customizing the CNN-SK model structure

**Fig 10:**
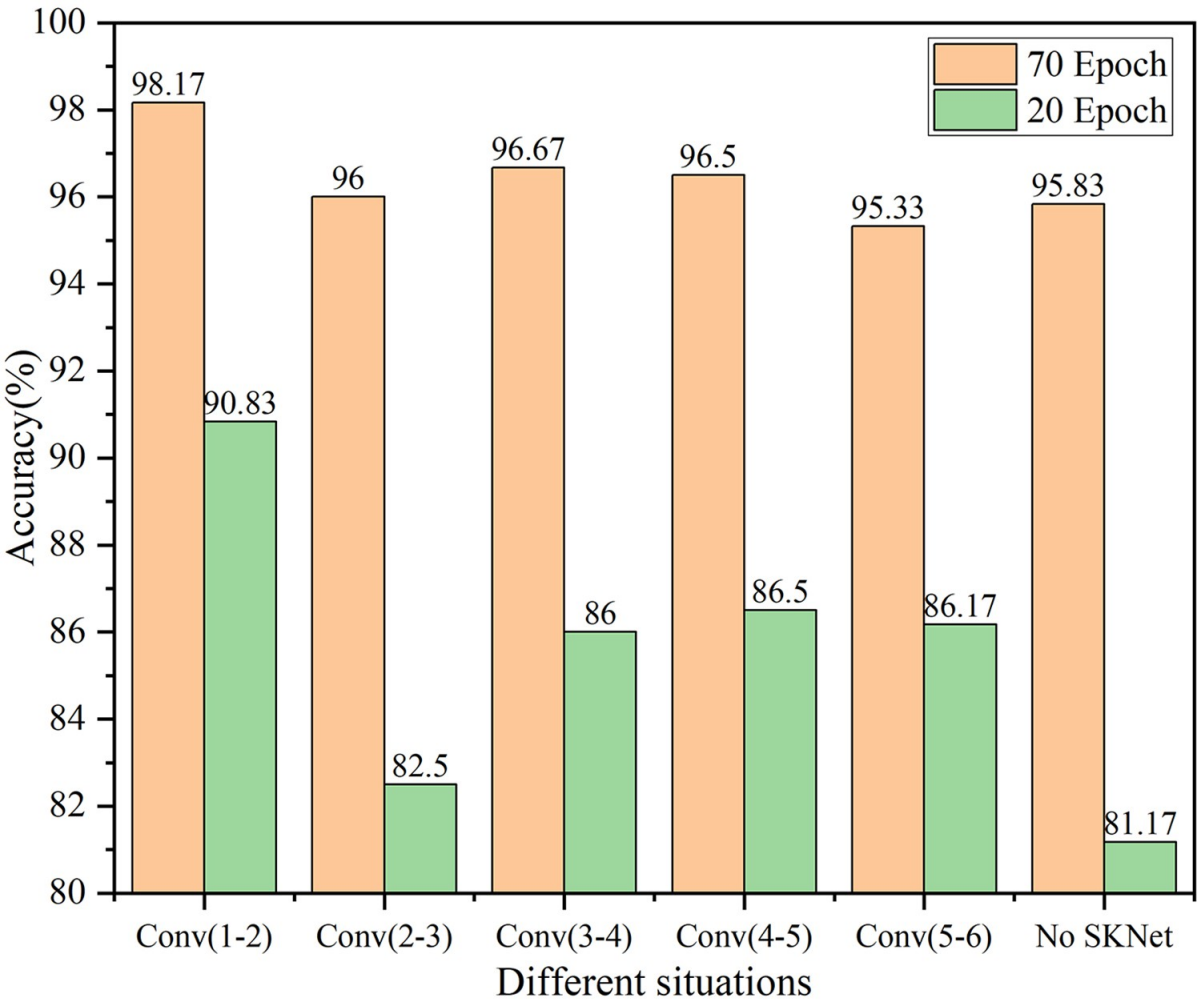
Accuracy curves obtained with different SKNet insertion locations during the validation process.

As mentioned in customizing lightweight CNN networks, the custom CNN consists of six convolutional layers. The SKNet is inserted between layer 1 and layer 2, between layer 2 and layer 3, between layer 3 and layer 4, between layer 4 and layer 5, and between layer 5 and layer 6. Fig 10 shows a comparison among the accuracies achieved on the validation set by the models with the SKNet inserted in the five location mentioned above, as well as the model to which no SKNet is added. The accuracies achieved at 70 and 20 epochs are measured to characterize the accuracy and convergence rate of each model.

**Fig 11:**
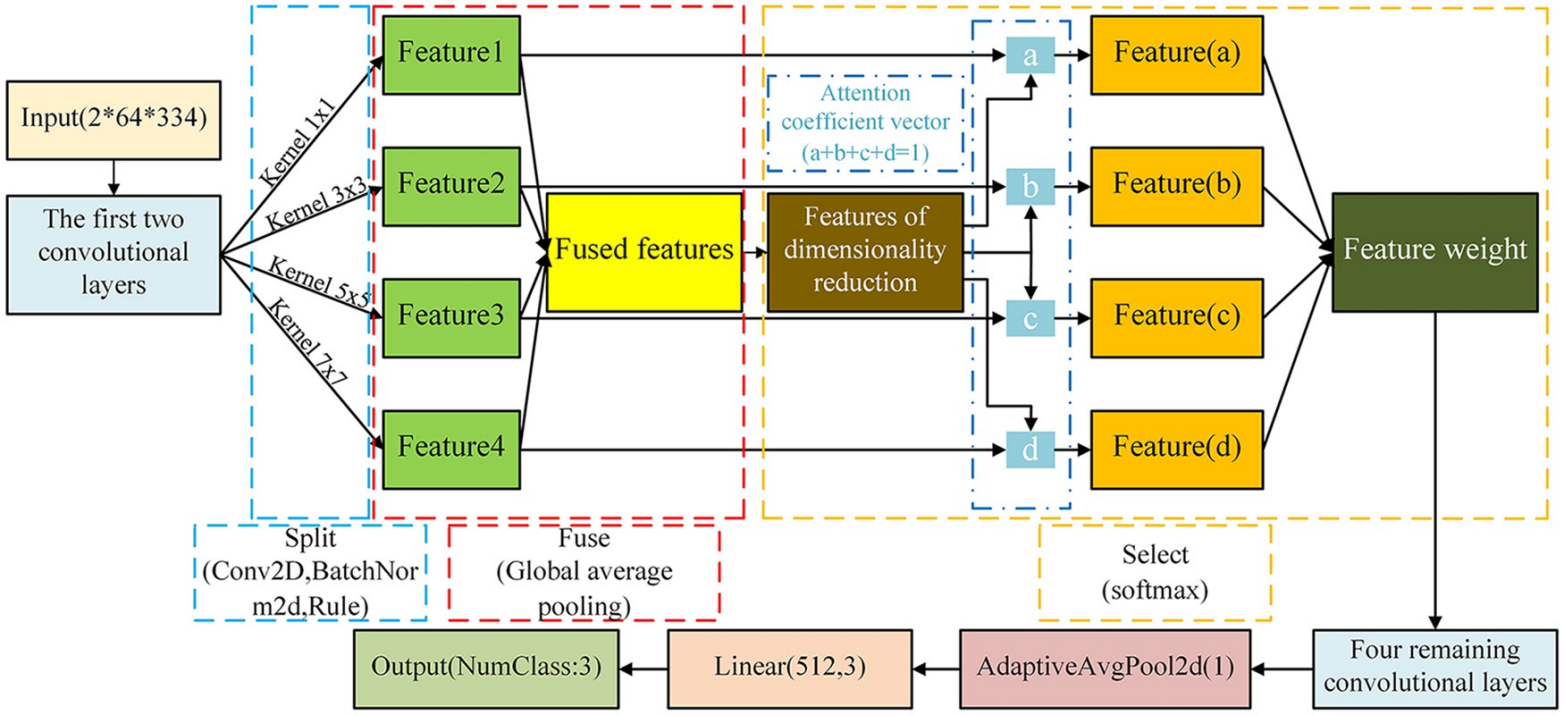
Customized CNN-SK structure.

As depicted in Fig 10, the inclusion of the attention mechanism results in enhanced model accuracy, and the degree of improvement is influenced by the placement of this mechanism. The accuracy values in Fig 10 clearly show that when the SKNet is inserted between layer 1 and layer 2, both the final recognition accuracy and the convergence rate of the model are optimal. On the basis of this observation, the structure of the custom CNN-SK model developed in this paper, as shown in Fig 11, involves passing the input data through the SKNet after the first-layer convolution. Initially, the data undergo feature extraction steps with four different kernel sizes, resulting in four distinct feature mappings. These four features are then combined to obtain global and integrated representations, which are used for weight selection. To minimize the number of computations, the aggregated features are downscaled. The resulting feature vectors are then individually convolved with the four attention coefficient vectors. This process forms feature selections that are reaggregated at various angles. Finally, Softmax penetration is applied to calculate the feature weights, which are then passed on to the next convolutional layer.

### Hyperparameter adjustment process of CNN-SK

**Fig 12:**
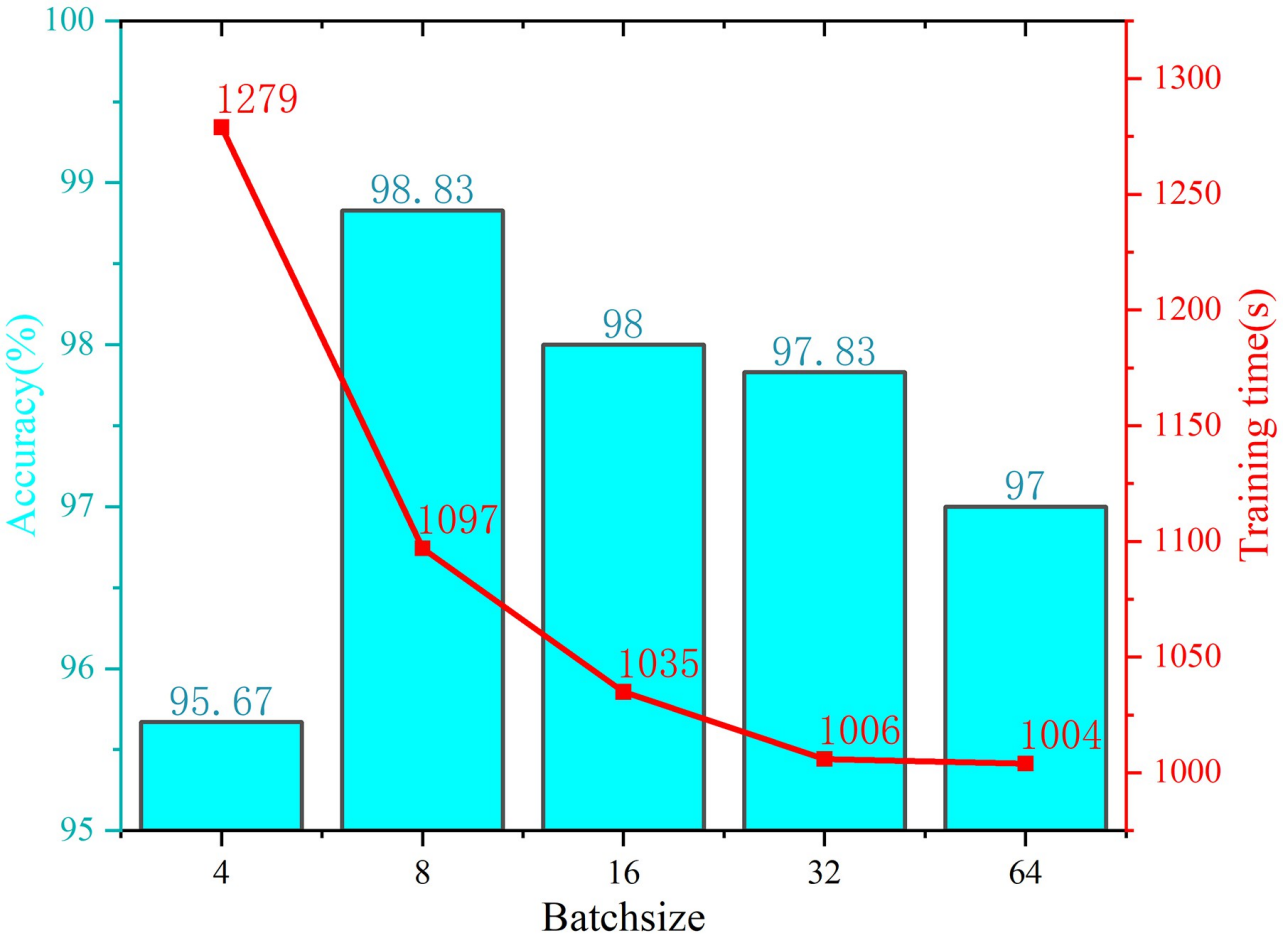
Batch size adjustment curve.

Batch size adjustment: The batch size plays a crucial role in determining the convergence and generalization capabilities of a model. A comparison is conducted among the model training accuracy rates achieved using batch sizes of 4, 8, 16, 32, and 64, as illustrated in Fig 12. The results indicate that adjusting the batch size has a discernible effect on the performance of the model. Notably, the figure shows that as the batch size increases, the running time of the model decreases, whereas the training accuracy initially increases but then decreases. The optimal batch size of 8 yields the highest verification accuracy. Consequently, for the purposes of this study, setting the batch size to 8 is deemed appropriate.

**Fig 13:**
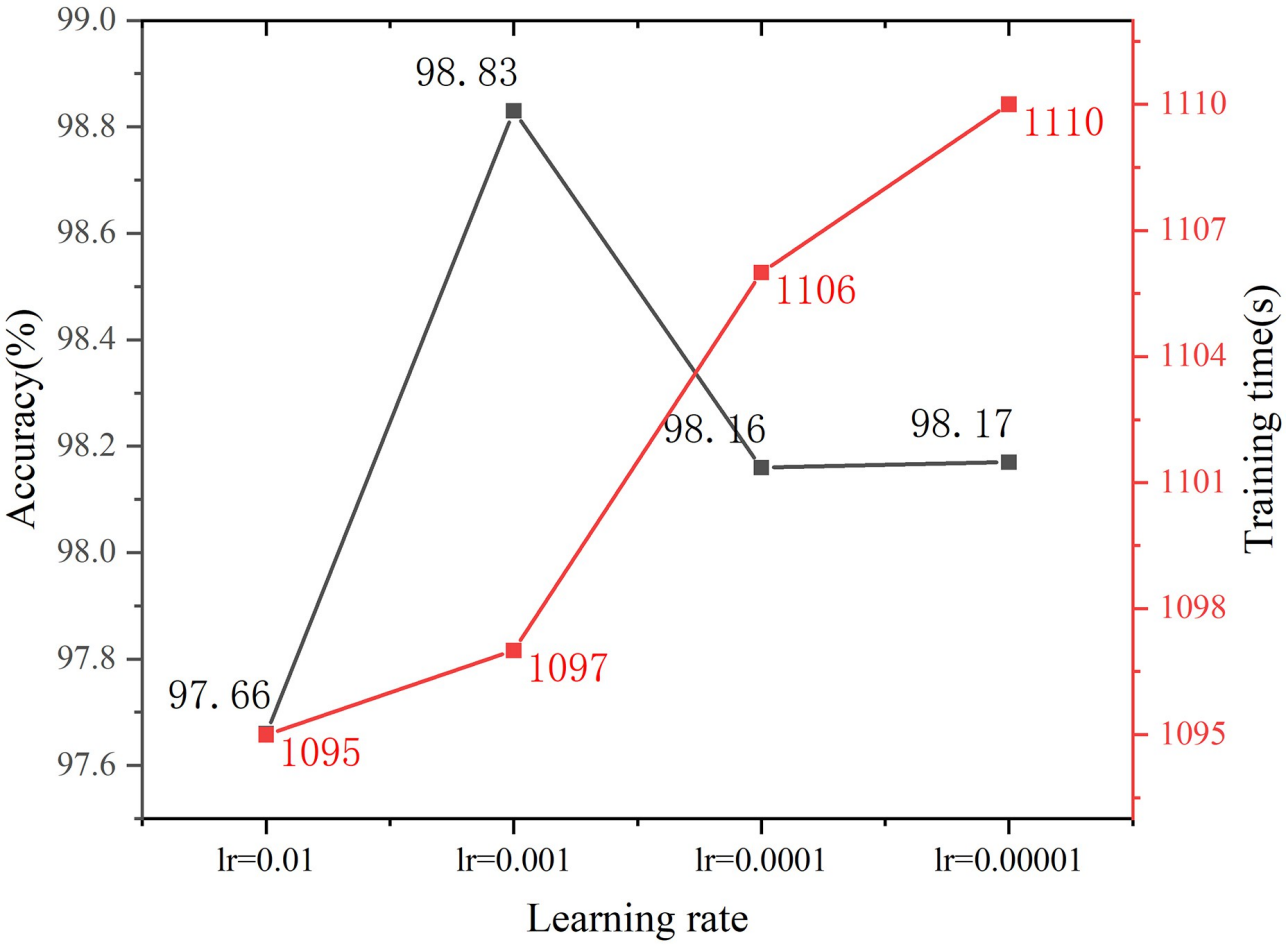
Learning rate adjustment curve.

Learning rate adjustment: The learning rate is a crucial factor for enhancing the training efficiency and performance of a model. To determine the optimal learning rate for the model, various learning rates, such as 10^−2^, 10^−3^, 10^−4^, and 10^−5^, are tested to compare the verification accuracies of the constructed models. The results, depicted in Fig 13, illustrate that as the learning rate decreases, the accuracy of the model initially improves before it decreases, whereas the training time gradually increases. Notably, at a learning rate of 10^−3^, the model achieves its highest accuracy with a shorter training time. Therefore, upon comprehensively considering these factors, the optimal learning rate is determined to be 10^−3^.

### Performance of CNN-SK model

A performance comparison involving the attention mechanism model reveals that the SKNet offers significant advantages when customizing the CNN model. To validate the efficacy of CNN-SK in penetration-through recognition, this study selects two widely used feature extraction networks, namely, VGG and AlexNet, for a comparison with CNN-SK. Additionally, to emphasize the lightweight nature of CNN-SK, we deliberately choose the even lighter LeNet network for the performance comparison. To assess the superior recognition capabilities of the proposed model in the domain of welding penetration identification, the VGG-SE and TF-CNN models [21, 22], which are closely related and highly regarded in this field, are chosen for an recognition accuracy comparison with CNN-SK. The research conducted in [21] marked the initial utilization of arc sound signals and CNNs for weld penetration state classification, thereby broadening the scope of CNNs in the field of intelligent welding monitoring. The investigation in [22] further integrated an attention mechanism into intelligent welding monitoring, underscoring the importance of comparing the recognition accuracy of their approach with that of the model in this study.

The comparison results are shown in Table 6. Table 6 reveals that the accuracy rates of CNN-SK, LeNet, VGG, AlexNet, TF-CNN, and VGG-SE are 98.83%, 92.33%, 94.17%, 95.50%, 98.20% (100 epochs), and 98.25% (100 epochs), respectively. Among these models, LeNet results in a shorter training time, a smaller memory footprint, and a lower number of floating-point operations (FLOPs) per batch than does CNN-SK. However, the final recognition performance of LeNet is significantly inferior to that of CNN-SK. On the other hand, both VGG and AlexNet fall short of CNN-SK in terms of all the measured metrics. This clearly demonstrates that CNN-SK achieves enhanced model accuracy without squandering computational resources.

**Table 6:** Comparison among the operational performances of models.

Model	Accuracy(%)	Training time(epoch = 70)	Params size(MB)	FLOPs
CNN-SK	98.83	21 min 03 s	15.12	2776778880
LeNet	92.33	20 min 05 s	7.95	914104320
VGG	94.17	28 min 30 s	128.21	107612577792
AlexNet	95.50	28 min 41 s	217.47	4686407680
TF-CNN	98.20	-	-	-
VGG-SE	98.25	-	-	-

**Fig 14:**
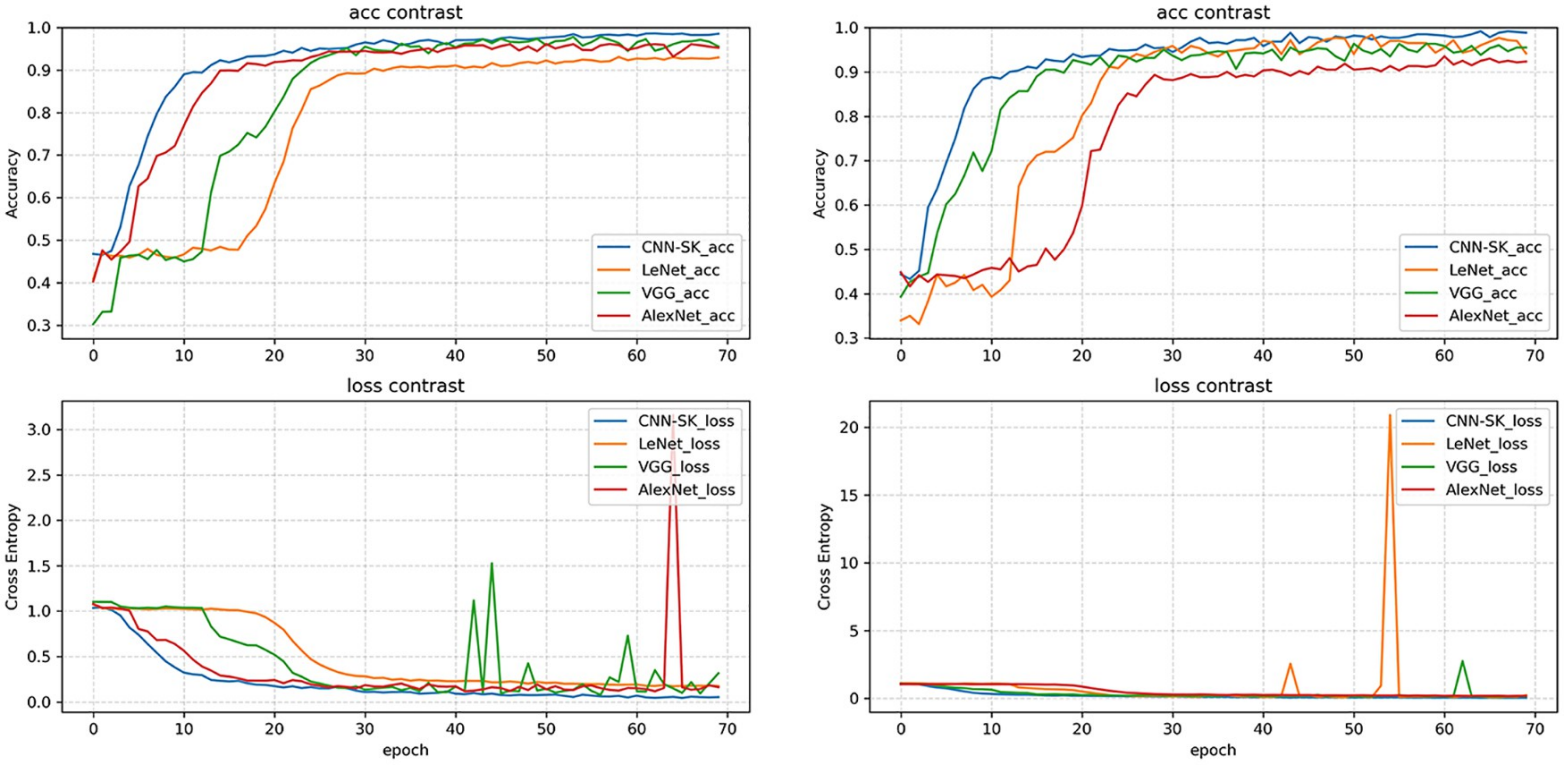
Accuracy and loss curves produced during the training and validation processes. (a) Training process. (b) Validation process.

Fig 14 compares the running performance of CNN-SK with that of other conventional models during the training and validation dataset, demonstrating that CNN-SK outperforms these models in terms of both its final accuracy and convergence speed. To synthesize the exceptional performance of CNN-SK, a three-dimensional confusion matrix of the penetration states is depicted in Table 7. This matrix enables an exploration of the model performance in terms of accuracy, precision, recall, the F1 score, and the weighted average, as expressed mathematically in Eqs (4–10). The accuracy rate reflects the proportion of correctly classified samples out of the total number of samples, serving as a comprehensive index for evaluating overall recognition performance. The precision rate indicates the ratio of correctly predicted positive cases to the total number of predicted positive cases. The recall rate represents the ratio of correctly predicted positive cases to the total number of actual positive cases. The F1 score represents the harmonized average of the precision and recall rates. The weighted average refers to a combination of metrics determined by considering the importance of each category in the dataset.

$$\begin{array}{c} \text{Accuracy}=(T00+T11+T22)/N \end{array}$$ (4)

$$\begin{array}{c} {\text{Precision}}_{i}=Tii/\left(Tii+Fji\right) \end{array}$$ (5)

$$\begin{array}{c} {\text{Recall}}_{i}=Tii/\left(Tii+Fij\right) \end{array}$$ (6)

$$\begin{array}{c} F1-{\text{score}}_{i}=\left(2\times Recall_{i}\times Precision_{i}\right)/\left(Recall_{i}+Precision_{i}\right) \end{array}$$ (7)

$$\begin{array}{c} \begin{array}{cc} \text{weighted}-{\text{average}}_{\text{Precision}}= & (Precision_{i}\times support0\\ & +Precision_{i}\times support1\\ & +Precision_{i}\times support3)/N \end{array} \end{array}$$ (8)

$$\begin{array}{c} \begin{array}{cc} \text{weighted}-{\text{average}}_{\text{Recall}}= & (Recall_{i}\times support0\\ & +Recall_{i}\times support1\\ & +Recall_{i}\times support3)/N \end{array} \end{array}$$ (9)

$$\begin{array}{c} \begin{array}{cc} \text{weighted}-{\text{average}}_{F1-\text{score}}= & (F1-score_{i}\times support0\\ & +F1-score_{i}\times support1\\ & +F1-score_{i}\times support3)/N \end{array} \end{array}$$ (10)

Where N is the total number of samples, support0, support1, and support2 represent the number of samples in each of the three categories. In this paper N = 300, support0 = 100, support1 = 100, support2 = 100.

**Table 7:** Confusion matrix of three penetration states.

Predicted Label	True Label		
	None penetration(0)	Full penetration(1)	Excessive penetration(2)
None penetration(0)	T00	F01	F02
Full penetration(1)	F10	T11	F12
Excessive penetration(2)	F20	F21	T22

**Fig 15:**
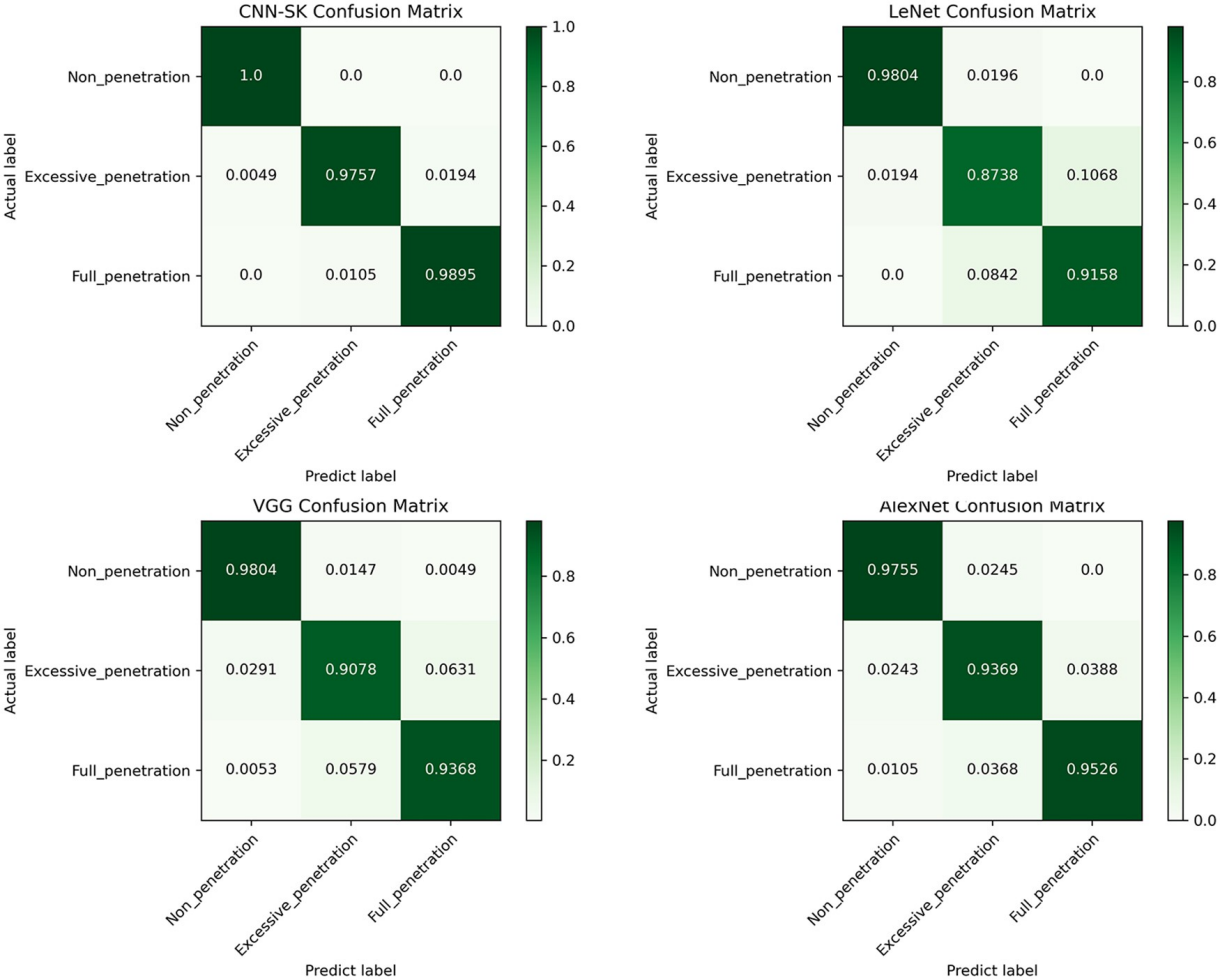
Confusion matrices for comparing CNN-SK with other conventional models.

Table 8 displays the precision, recall, F1-score, and weighted average metrics produced by the four models on test dataset. The corresponding confusion matrices constructed for these models are illustrated in Fig 15, where a higher concentration of diagonal elements near 100% signifies enhanced classification accuracy within the corresponding category. Notably, CNN-SK demonstrates superior prediction accuracy to those of the other models across all states, with LeNet exhibiting comparatively weaker recognition performance (some metrics fall below 90%). Conversely, CNN-SK excels at recognizing each category, underscoring its exceptional classification capabilities. The CNN-SK network developed in this study incorporates a dynamic selection mechanism, enabling the model to comprehend Mel spectrum features at various levels and extract information from diverse perspectives. Through an analysis of the multidirectional attributes of the Mel spectrum, the proposed model shows outstanding recognition performance even with a shallow architecture, limited operational parameters, and a small sample size.

**Table 8:** Precision, recall, F1-score and weighted average metrics achieved by the four models on test dataset.

Model		Weighted average	Label		
			None penetration	Full penetration	Excessive penetration
CNN-SK	Precision	0.9884	0.9951	0.9901	0.9792
	Recall	0.9883	1.0000	0.9757	0.9895
	F1-score	0.9883	0.9976	0.9829	0.9843
LeNet [40]	Precision	0.9235	0.9804	0.9000	0.8878
	Recall	0.9233	0.9804	0.8738	0.9158
	F1-score	0.9233	0.9804	0.8867	0.9016
VGG [41]	Precision	0.9415	0.9662	0.9303	0.9271
	Recall	0.9417	0.9804	0.9078	0.9368
	F1-score	0.9415	0.9732	0.9189	0.9319
AlexNet [42]	Precision	0.9549	0.9660	0.9415	0.9577
	Recall	0.9550	0.9755	0.9369	0.9526
	F1-score	0.9550	0.9707	0.9392	0.9551

### Visualization of the feature extraction process implemented by CNN-SK

**Fig 16:**
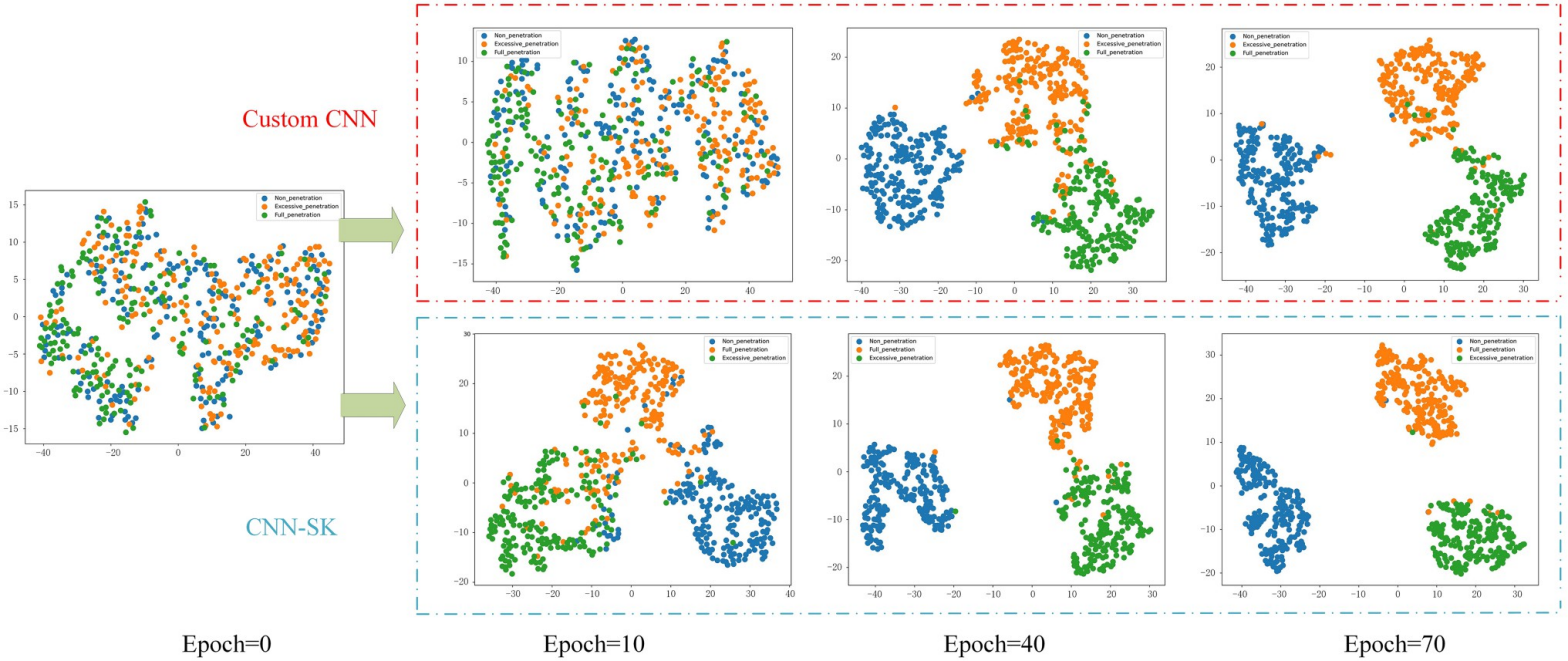
Comparisons among the t-SNE downscaling results of different models.

To further illustrate the benefits of the feature extraction process used by CNN-SK and the effectiveness of the SKNet in custom CNNs, the t-distributed stochastic neighbor embedding (t-SNE) down sampling method is utilized to visualize the classification process of the model in the form of a scatter plot. The comparisons between the classification performances achieved by the custom CNN and CNN-SK models on the validation set at 0, 10, 40, and 70 iterations are depicted in Fig 16. This approach allows for a clear demonstration of the effectiveness of the classification models.

As depicted in Fig 16, the data distribution of the validation set appears to be random, with overlapping feature components for each category, making it challenging to differentiate between the included categories. However, after 10 model iterations, a noticeable feature separation trend among the three penetration states is observed, with the CNN-SK model exhibiting a more pronounced effect in terms of separating features than the customized CNN model does. At this stage, the outlines of the features belonging to each category begin to emerge. After 40 iterations, the feature separation phenomenon becomes increasingly evident for both models. After 70 iterations, both models produce relatively obvious class segmentation regions. However, many misclassified samples remain for the custom CNN, and only a few cases of none penetration are misclassified by CNN-SK as belonging to the other two categories. This observation highlights the impressive feature extraction ability of CNN-SK and provides further evidence of the effectiveness of the SKNet in customized CNN models.

## Conclusions

In this study, a novel CNN-SK penetration recognition network was devised to evaluate penetration statuses during the CMT welding procedure by analyzing arc sound signals. The effectiveness of this methodology was confirmed through empirical studies, culminating in the following findings.

1. The likelihood of the arc sounds generated during CMT welding is most prominent in the 0–2 kHz frequency range. By utilizing the sensitivity of the Mel filter to low-frequency signals, the Mel spectrum can efficiently highlight the unique characteristics of arc sound signals.
2. A 6-layer CNN model is suitable for lightweight applications, although it may sacrifice recognition efficiency. The experimental findings indicated that incorporating the dynamic SKNet selection mechanism significantly enhanced the performance of the model. The most effective location for integrating this mechanism is within the 1st or 2nd convolutional layer. Consequently, a CNN-SK network was developed, and it demonstrated efficacy in terms of accurately identifying weld penetration states.
3. The CNN-SK weld penetration recognition model demonstrated a recognition accuracy of 98.83% for the three penetration states, surpassing the performance of other sophisticated models, such as VGG, AlexNet, and LeNet. This superior accuracy can be attributed to the incorporation of distinct nuclei within the CNN-SK model, enabling it to more precisely identify penetration than its predecessors could.

In this research, a CNN-SK model for recognizing weld penetration was developed using the Mel spectrum of CMT arc sound signals, resulting in a successful recognition outcome. However, limitations exist in this study, notably concerning the collection of structural arc sound signals in an optimal environment. Future endeavors will need to address the challenges related to deploying lightweight models on microcontrollers. Furthermore, incorporating diverse mechanical operational noises and mixed noise signals derived from real-world settings into experimental data poses a significant challenge in terms of determining whether the model can maintain high recognition accuracy on such a dataset.

## Supporting information

S1 File Dataset. (DOCX)
