# Supplementary material for: Construction of a CNN-SK weld penetration recognition model based on the Mel spectrum of a CMT arc sound signal
Source: PLoS One. 2024 Nov 25;19(11):e0311119. doi: 10.1371/journal.pone.0311119 (PMC11588216; doi:10.1371/journal.pone.0311119)
Supplement: S1 File — (DOCX) [file pone.0311119.s001.docx]

**Supporting information**

In our experiment, a series of welding experiments were carried out to analyze the relationship between welding defects and arc sound signals.

The datasets can be downloaded from the following link: https://github.com/ZWL58/data/tree/master.
